# Spatial ultrasound modulation by digitally controlling microbubble arrays

**DOI:** 10.1038/s41467-020-18347-2

**Published:** 2020-09-10

**Authors:** Zhichao Ma, Kai Melde, Athanasios G. Athanassiadis, Michael Schau, Harald Richter, Tian Qiu, Peer Fischer

**Affiliations:** 1 Max Planck Institute for Intelligent Systems, Heisenbergstr. 3, 70569 Stuttgart, Germany; 2 Institut für Mikroelektronik Stuttgart, Allmandring 30a, 70569 Stuttgart, Germany; 3 Institute of Physical Chemistry, University of Stuttgart, Pfaffenwaldring 55, 70569 Stuttgart, Germany

**Keywords:** Mechanical engineering, Acoustics, Fluid dynamics

## Abstract

Acoustic waves, capable of transmitting through optically opaque objects, have been widely used in biomedical imaging, industrial sensing and particle manipulation. High-fidelity wave front shaping is essential to further improve performance in these applications. An acoustic analog to the successful spatial light modulator (SLM) in optics would be highly desirable. To date there have been no techniques shown that provide effective and dynamic modulation of a sound wave and which also support scale-up to a high number of individually addressable pixels. In the present study, we introduce a dynamic spatial ultrasound modulator (SUM), which dynamically reshapes incident plane waves into complex acoustic images. Its transmission function is set with a digitally generated pattern of microbubbles controlled by a complementary metal–oxide–semiconductor (CMOS) chip, which results in a binary amplitude acoustic hologram. We employ this device to project sequentially changing acoustic images and demonstrate the first dynamic parallel assembly of microparticles using a SUM.

## Abstract (web summary)

The authors introduce a dynamic spatial ultrasound modulator, based on digitally generated patterns of microbubbles controlled by a complementary metal–oxide–semiconductor (CMOS) chip. They achieve reshaping of incident plane waves into complex acoustic images and demonstrate dynamic parallel assembly of microparticles.

## Introduction

A fundamental property of waves is that they diffract. Spatially modifying the phase or amplitude of an incident wave can be used to focus the wave or to form a diffraction image with the desired intensity distribution. While it is possible to dynamically modify the phase and amplitude of light waves with the help of a spatial light modulator (SLM)^1,2^, it has proven challenging to similarly control sound waves. There exist a variety of methods to dynamically tune the phase and amplitude of light through techniques, including phase retardation in liquid crystals^3^, geometric-phase tuning via metasurfaces^4,5^, and binary switching of reflected amplitude via micro-electromechanical systems^6^. Acoustic waves possess no polarization and show no or little dispersion from the low audible kHz to the very high MHz ultrasound frequencies^7^, which considerably complicates the realization of a spatial modulator for sound waves analogous to an SLM.

Recently, it has been demonstrated that static-phase plates, or holograms^8^, can modify an ultrasound field at high resolution with more than 10,000 pixels across the wavefront. This considerably increases the complexity of the projected static ultrasound fields, which has enabled first demonstrations of acoustic fabrication^9^ and the assembly of cells^10^ into designed patterns, beam steering^11^, and the compensation of wavefront aberration in transcranial focusing of ultrasound^12^. The ability to dynamically update and adjust these complex ultrasound fields with the aid of a high-resolution spatial ultrasound modulator (SUM), would present a major advance for these and related applications, which include medical imaging deep inside the body^13,14^, nondestructive testing of opaque solids^15^, the manipulation of submicron particles^16,17^, biological cells^18,19^, and even centimeter-sized objects^20^.

The realm of audible acoustics has seen some notable developments in this regard. Ma et al. demonstrated a metasurface of membrane-type resonators to dynamically control and reshape a reverberating sound field in a room^21^. Another system reported by Tian et al. used an array of tunable Helmholtz resonators to steer and focus transmitted acoustic waves^22^. The large wavelengths of audible acoustic waves relative to the region of interest result in a differently scaled problem with low degrees of freedom, where a small number of larger actuators is sufficient. This is contrary to the previously mentioned applications of high-frequency ultrasound, which benefit from large numbers of much smaller pixels. The conventional device for ultrasound beam shaping is the phased array transducer (PAT)^23^, which uses many individually controllable sound emitters to directly generate arbitrary and dynamically tunable wavefronts via superposition. PATs have been shown to efficiently implement dynamic holograms and project the complex trapping fields that enabled acoustic tweezers^24^. Taking advantage of their fast update rate, PATs can generate multiple traps via time multiplexing^25^ or relocate a single trap occupied by a particle at high speed of several meters per second^26^. However, the complexity in the driving circuit limits the total number of PAT pixels to <1000. This is well below the number of elements that would be needed to enable sophisticated control of an ultrasound wave. Further, having the sound wave generation and the shaping of the wave in the same device increases the complexity and limits the development of high-power devices with many degrees of freedom. Spatial ultrasound modulation could solve this problem, as it would decouple the generation of the ultrasound wave from its modification, and thus would permit the use of an optimized single-element transducer.

Here, we introduce a dynamic SUM based on digitally generated microbubbles on a complementary metal–oxide–semiconductor (CMOS) chip surface. Due to the strong acoustic impedance mismatch between a gas bubble and the surrounding liquid^27,28^, we modify the transmission of an acoustic wave with programmable microbubbles, in analogy to the digital mirror device (DMD) for spatial light modulation^29^. We write binary amplitude holograms with 10,000 digitally addressable microbubble pixels on the CMOS chip surface within 12 s through water electrolysis. Between frames, the SUM surface is mechanically reset, which allows us to realize the first high-resolution animation of sequential acoustic images. We demonstrate the versatility of a SUM by assembling microparticles into complex shapes.

## Results

### Principle of spatial ultrasound modulation via a microbubble array

Implementing a dynamically reprogrammable phase plate similar to the static acoustic hologram^8^ is an engineering challenge. The obvious approach through, e.g., deformable surfaces^30,31^, requires the integration of many actuators with spacing and displacements at the ultrasound wavelength scale. Alternatively, controlling dispersion could efficiently modulate the phase of an ultrasound wave, but no suitable material or meta-material concept has been found to date. Amplitude modulation promises a more viable solution instead of phase^29^. Though a binary amplitude hologram contains only two states for each element, which decreases its information capacity compared to multiple-level phase modulation, it could still afford complex image generation, simply by providing many more elements in total^29^.

**Fig. 1:**
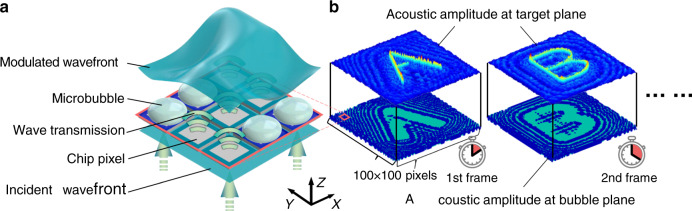
Schematic of the spatial ultrasound modulation (SUM) based on microbubble patterns. **a** A microbubble can effectively block the acoustic transmission since its acoustic impedance differs significantly from the surrounding liquid. A pattern of microbubbles can therefore spatially modulate the incident plane acoustic wave and give it a complex wavefront. **b** The microbubble pattern encodes the binary amplitude kinoform (hologram) of the target acoustic field. Refreshing the microbubble pattern enables dynamic spatial ultrasound modulation.

Due to the significant acoustic impedance mismatch between gas and liquid, a thin layer of air in liquid can effectively stop ultrasound, even when its thickness is less than the acoustic wavelength. A microbubble can thus serve as a local sound blocker. A pattern of microbubbles in the path of an ultrasound wave should, therefore, impart a corresponding amplitude pattern onto the wavefront of the acoustic field, which is the operating principle of our SUM, as shown in Fig. 1a. Patterning a large number of microbubbles enables the on-demand shaping of an acoustic field’s amplitude distribution (Fig. 1b). Moreover, the dynamic control of the microbubble pattern enables dynamic spatial acoustic modulation. Based on this concept, our dynamic spatial ultrasound modulator (SUM) generates reconfigurable microbubble patterns.

For example, even a 20-μm gas layer leads to a negligibly small transmission coefficient (on the order of 10^−7^), considering a 10-MHz acoustic wave (wavelength 150 µm). This can be seen from the power transmission coefficient for an acoustic wave at normal incidence through a plain layer^32^:

1 $$C_T = \frac{1}{{\xi ^2\sin ^2\left( {k_L\delta } \right) + 1}},$$

2 $$\xi = \frac{1}{2}\left| {\frac{{Z_L}}{{Z_M}} - \frac{{Z_M}}{{Z_L}}} \right| = \frac{1}{2}\left| {\frac{{\rho _Lc_L}}{{\rho _Mc_M}} - \frac{{\rho _Mc_M}}{{\rho _Lc_L}}} \right|,$$

where *δ* and *k*_*L*_ are the layer thickness and wavenumber in the layer material, respectively; *Z*, *ρ*, and *c* are acoustic impedance, density, and speed of sound, respectively; the subscripts *L* and *M* indicate the layer and the surrounding host medium. The sound speed in water (*ρ*_*M*_ ~ 1000 kg m^−3^) is *c*_*M*_ ~ 1500 m s^−1^ and in air *c*_*L*_ ~ 343 m s^−1^ at atmospheric pressure (*ρ*_*L*_ ~ 1.23 kg m^−3^). As the ratio of acoustic impedances increases, the wave is increasingly reflected at the interface, and therefore, less energy is transmitted through the layer. Since air blocks ultrasound so well, we now need to find a way to generate programmable on-demand microbubble patterns.

**Fig. 2:**
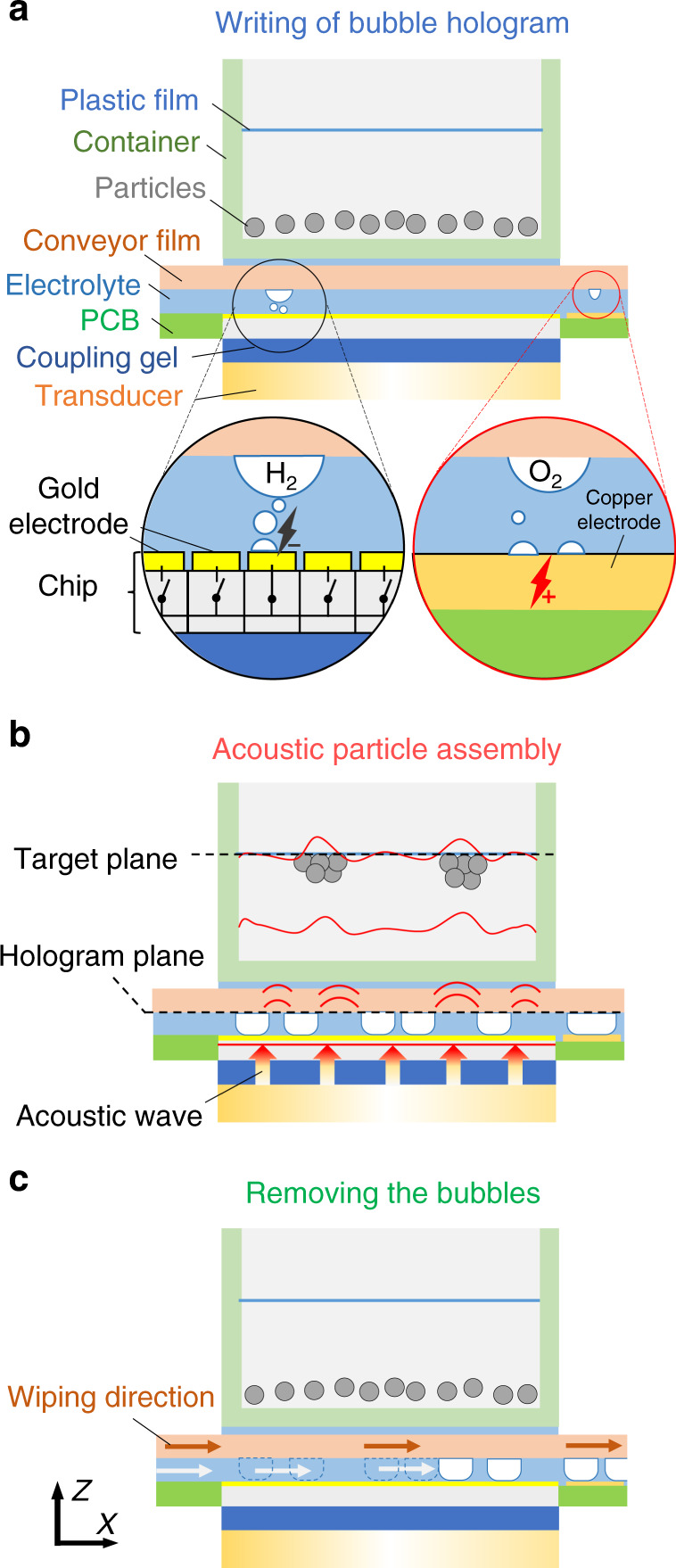
Schematic of the stepwise actuation of the rewritable acoustic hologram. **a** A bubble pattern is generated on-demand by a complementary metal–oxide–semiconductor (CMOS) chip with 100 × 100 electronically addressable pixels. A controlled current at each pixel causes electrolysis. **b** An ultrasonic transducer generates a plane acoustic wave that transmits through the chip. The presence of a microbubble locally blocks the acoustic wave. Thus, a spatially modulated wavefront is generated that represents a binary amplitude hologram. The wave passes only through the bubble-free regions. The modulated acoustic wave propagates and diffracts to form an acoustic image at the target plane. Suspended particles are concentrated in areas of high acoustic amplitude by the acoustic radiation force. **c** A conveyor-driven polymer film removes the bubble pattern, and the cycle restarts.

Our SUM device architecture consists of a CMOS chip placed on top of an acoustic transducer, as shown in Fig. 2. A liquid film of electrolyte is sandwiched between the chip surface and a conveyor film. The CMOS chip surface has 10,000 individually addressable electrodes (70 μm by 70 μm gold pads in a 100 µm by 100-µm raster). Positioned next to the chip is a copper electrode, which serves as the anode. A switchable DC power supply provides a potential difference between the copper electrode (+5 V) and the 10,000 gold electrode pads of the CMOS chip. Once the DC power is switched to a CMOS pixel, the electrolysis of the surrounding water solution generates hydrogen and oxygen gas, respectively, at the gold and copper electrodes. As we will see below, the current is controlled to define the size of the microbubbles.

To generate a target acoustic field, we first compute a binary amplitude hologram^8^, which is a binary transmission function that can be directly translated into a pattern of microbubbles. The CMOS chip then generates microbubbles according to this pattern. Each microbubble corresponds to a location of zero ultrasound transmission (Fig. 2a). After the bubble generation is completed, the transducer is turned on (Fig. 2b), and the acoustic wave transmits through the SUM and is locally blocked at the pixels that are covered by a microbubble. The remainder of the wavefront propagates into the upper container and diffracts to form the target sound pressure distribution. To visualize the pressure field at the target plane, we introduced submillimeter PDMS particles suspended in water, which then assemble into the shape of the projected sound pressure image. To conclude the sequence and prepare the SUM for the next frame, the microbubbles are cleared by horizontally translating a conveyor film (Fig. 2c), which drags the bubbles out of the device. The complete modulation process is shown in the Supplementary Movie 1.

### Microbubble generation

**Fig. 3:**
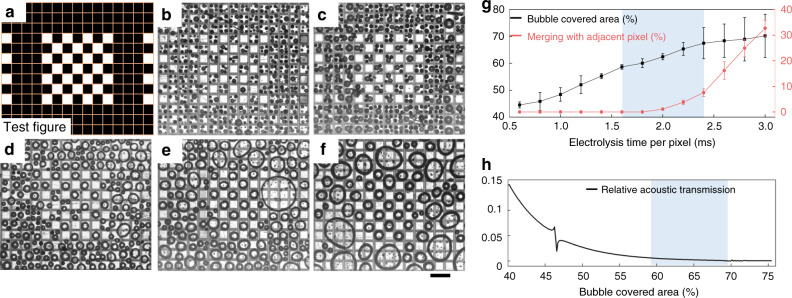
Bubble evolution as a function of the time for electrolysis. **a** The test pattern, where the dark pixels mark areas that receive current, and white pixels that do not. Extending the duration of the electrolysis from (**b**) 0.6 ms, (**c**) 0.8 ms, (**d**) 1.6 ms, (**e**) 2.4 ms to (**f**) 2.8 ms, increases the size and the coverage of the bubbles. Merging of growing bubbles with those at adjacent pixels (**g**). The optimal electrolysis time range for the spatial ultrasound modulation (SUM) (marked blue) is thus somewhere between **d** and **e**, i.e., between 1.6 ms and 2.4 ms. **h** Simulation shows that this coverage results in the blocking of 99% of the acoustic intensity. The scale bar is 200 μm.

The SUM generates a pattern of microbubbles on the surface of the CMOS chip by the electrolysis of water. The microbubble coverage has to be large enough to ensure that the acoustic wave is blocked at the location of the electrode. As the potential difference between the anode and the cathode is constant (5 V), the microbubble volume depends on the time the current flows. The size of the microbubbles as a function of the time of the electrolysis (0.6, 0.8, 1.6, 2.4, and 2.8 ms) is shown in Fig. 3. The area (*X*–*Y* plane) covered by microbubbles increases with the duration of the electrolysis. An adequate microbubble volume also ensures that the bubble is trapped between the conveyer film and the chip surface. The adherence to the solid surfaces appears quite strong and retains the microbubbles against buoyancy even when the device is turned to a vertical orientation^33^. This suggests that the operability of our SUM is independent of its orientation, as shown in Supplementary Fig. 1. However, as the microbubbles grow, neighboring bubbles can fuse, which is shown in Fig. 3g. This distorts the microbubble pattern because the resulting merged bubbles adopt a spherical shape due to surface tension. We empirically determined that a flow of current between 1.6 and 2.4 ms, marked in blue in Fig. 3g, maximizes the bubble coverage while keeping the fusion of bubbles low.

Figure 3h shows the simulated relative acoustic transmission coefficient for different bubble coverages across a single pixel. The relative acoustic transmission coefficient is the ratio of the acoustic intensity transmitted through a bubble covered pixel versus an uncovered pixel. It can be seen how the selected bubble coverage (marked blue), resulting from the selected electrolysis time, effectively blocks 99% of the incident acoustic intensity. It should be noted that the applied acoustic frequency (10 MHz) is far above the fundamental resonant frequency (on the order of 100 kHz) for 10-μm-sized microbubbles in water^34^. Thus, the bubble vibration excited by the incident acoustic waves is negligible^35^. Accordingly, we do not observe bubble motion even when the intensity at the transducer reaches about 5 W cm^−2^, which is sufficiently high for microparticle assembly and manipulation.

### Binary amplitude acoustic hologram

**Fig. 4:**
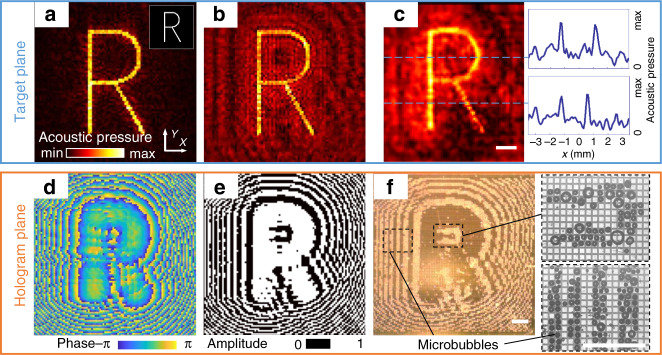
Performance of the 10,000-element spatial ultrasound modulator. Calculation of the phase hologram for the target image (**a**) and the corresponding phase front in the plane of the hologram (**d**). Calculations are based on the iterative angular spectrum method, and the phase distribution at the hologram plane is modified upon the back-propagation from the target acoustic amplitude field (top right inset). Comparison of the same target in a binary amplitude hologram (**b**, **e**). The experimental result showing the hydrophone scan (**c**) of the acoustic field in the target plane, which is generated by the bubble array in the hologram plane of the SUM (**f**). All scale bars are 1 mm.

For each acoustic image, the microbubble pattern is pre-calculated as a binary amplitude acoustic hologram, consisting of pixels with an amplitude of zero or one. Similar to a phase hologram (Fig. 4a, d)^8^, the binary amplitude hologram (Fig. 4b, e) can also be optimized using the iterative angular spectrum approach (IASA). In this special case, however, the phase distribution in the hologram plane is at each step converted to a binary amplitude distribution with a fixed phase. An average phase value is obtained from the back-propagated target image. The hologram pixels, whose original phase is within the range of ±π/2 from this average value, are set to an amplitude of one, and the remaining pixels are set to zero. The algorithm typically converges in <30 iterations.

Figure 4 shows simulations of reconstructed sound fields, and their corresponding holograms for a phase hologram (panels a, d) and binary amplitude hologram (b, e) encoding the letter “R”. Since the pixels in binary amplitude holography only have two states (Fig. 4e), they naturally provide much less information density than phase holograms which provide almost a continuous modulation over a range of 2π (Fig. 4d). This results in an elevated background noise that can be seen when comparing Fig. 4b with 4a. On the SUM chip surface, microbubbles replicate the zero-amplitude pixel pattern designed by the binary amplitude holography (Fig. 4f). The 10-MHz transducer emits an acoustic plane wave that transmits through the chip layer and then reaches the microbubble layer. The wave is blocked by each bubble and therefore modulated in amplitude. Where there is no microbubble, the wave transmits and diffracts to form the calculated acoustic image in the target plane (Fig. 4c). To demonstrate that the SUM can be used to project changing acoustic fields, we show a movie of the corresponding hydrophone scans in the Supplementary Movie 2. In this video, each frame was formed in 15 s, and the resulting field was raster-scanned by a needle hydrophone before clearing the bubble pattern and creating the next frame.

### Dynamic microparticle manipulation based on SUM

**Fig. 5:**
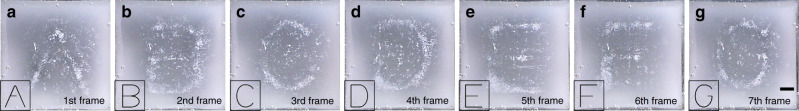
Particle patterning using the spatial ultrasound modulator (SUM). **a**–**g** Multiple frames of particles patterned by the SUM acoustic field in the form of the letters “A” to “G”. The scale bar is 1 mm. The dynamic process is shown in the Supplementary Movie 3.

Acoustic particle manipulation is an emerging technique with promising applications in fabrication^36^ and biomedical engineering^18^. To date, however, methods for dynamic and parallel manipulation have been limited to few particles^25^ or highly symmetric arrangements^37^. As shown in Fig. 5, the present SUM is capable of dynamically assembling microparticles into arbitrary target patterns. We use PDMS particles, which have a positive acoustic contrast in water. Thus, the acoustic radiation force on these particles will push them toward areas of high acoustic amplitudes. For each acoustic image, it takes around 12 s to write the microbubble hologram, when each pixel is sequentially addressed. Afterward, the transducer is turned on for 15 s, generating ultrasound waves, which are modulated by the SUM and propagate to form the acoustic image in the target plane, where the PDMS particles aggregate into the corresponding shape. After each assembly step, the transducer is turned off, and a motorized film mechanically “wipes” the microbubbles off the chip surface. In one experiment, the sequence of microbubble writing, particle assembly and bubble removal is repeated seven times to sequentially assembly the particles in the shape of the letters “A” to “G”. A video of this dynamic microparticle manipulation is shown in Supplementary Movie 3.

## Discussion

In summary, we demonstrate the first dynamic SUM, which can be used to generate arbitrary images out of sound. The SUM has 10,000 active elements that are digitally controlled to form microbubbles via electrolysis. We show that the SUM can generate binary amplitude transmission holograms. Hydrophone scans of the projected ultrasound fields are in excellent agreement with simulation results. The projected acoustic fields can be updated and used to assemble microparticles in pre-defined shapes. Currently, the elements of the chip are sequentially addressed, which leads to relatively slow update cycles, but parallel pixel addressing^38^ is expected to drastically increase the refresh rate. To meet the requirements of portable biomedical devices, the bubble removal method can be implemented by other means, e.g., forced fluid flow of the electrolyte or on-chip reversal of the electrolysis^39^. Future work should explore multilevel amplitude or phase control of sound waves exploiting the resonant behavior of the microbubbles at specifically controlled sizes^40–43^. Spatial ultrasound modulators extend the capabilities of ultrasound applications and will be essential for medical imaging^13,44^, nondestructive testing^15^, holographic acoustic tweezers^8,25^, transcranial ultrasonic focusing^12^, acoustic fabrication^9^ and cell assembly^10^.

## Methods

### The CMOS chip

The CMOS chip consists of an array of 100 by 100 gold electrodes with a size of 70 µm by 70 µm. Under each electrode, a CMOS transmission gate connects the electrode to a vertical wire. Outside the electrode array, additional transmission gate switches collect the column wires into eight global wires, which lead to the chip pads and can be accessed from the outside of the chip. Two shift register chains, respectively, for row and column select, are fed by a digital driving signal to control the transmission gate groups. The chip is driven by a commercial microcontroller board (Arduino Mega 2560), which is loaded with the codes for chip electrodes addressing and electrolysis voltage switching. The thickness between the conveyor film and the chip surface is estimated to be 20 μm. A 2-μL electrolyte droplet is squeezed between the conveyor film and the chip surface under the experimental conditions, whose spread area is measured as 1 cm^2^.

The chip was produced by a classical 0.8-µm channel length CMOS technology. This p-well technology incorporates local oxidation of silicon device isolation, a single polysilicon layer as the gate electrode, and two Aluminum layers for interconnects with a total of 15 optical lithography steps. In addition, two lithography steps specialized post-processing was used for the gold electrodes^45^.

### Hydrophone scan of target acoustic field

The acoustic pressure field is mapped by hydrophone scanning. The transducer and the chip are immersed in a tank containing the electrolyte (80 mg mL^−1^ aqueous K_2_SO_4_ solution). The wiring of the PCB board is waterproofed with a cured polydimethylsiloxane (PDMS) covering. The bottom of the chip is placed in contact with the transducer. A 10-MHz AC signal with 5 Vpp amplitude is applied to the transducer (I3-1008-S-SU, ultrasound aperture 11 mm, Olympus Corporation, Japan). The generated acoustic waves transmit through the chip containing the microbubble pattern. The needle hydrophone (0.2 mm diameter, Precision Acoustics Ltd., UK) measurement across the imaging plane scans each point for 0.1 s, during which the signal from the hydrophone is amplified and filtered by a lock-in amplifier (Zurich Instruments, Switzerland). The scan area is 60–100 mm^2^, with a lateral resolution of 0.08–0.1 mm. A typical scan is completed in 30–60 min.

### Acoustic simulations

To simulate the transmission of the acoustic wave through the bubble layer, a finite element method (FEM)-based numerical simulation was conducted using COMSOL Multiphysics 5.3 acoustic-solid interaction module. The modeling schematic is shown in Supplementary Fig. 2. Briefly, a domain defined with gas properties simulates the gas bubble sandwiched between two solid interfaces. It is immersed in a cuboid domain of water. Close to the gas bubble, a cuboid domain of silicon is defined to simulate the chip. A 10-MHz vibration is located at the bottom surface of the chip. The acoustic wave transmits through the silicon chip, the gas bubble layer, and the water, and its far-field intensity is calculated. The remaining exposed boundaries are defined as symmetric boundaries.

### Microparticle patterning

PDMS microparticles are generated by homogenizing 10:1 weight ratio pre-polymer and curing agent (Sylgard184 Silicone Elastomer Kit, Dow Corning Corp., Freeland, MI) in 70 °C deionized water for 1 h. The setup used for the patterning of microparticles is shown in Supplementary Fig. 3. The 10-MHz AC signal from a function generator is amplified to 5 W by a power amplifier and applied to the transducer. The chip is placed on top of the transducer with a thin layer of glycerol for acoustic coupling. The aqueous K_2_SO_4_ solution is pipetted onto the chip surface, and then a plastic thin film is sandwiched between the chip and a 3D-printed container. To refresh the SUM and remove all microbubbles, the thin film is horizontally dragged across the chip surface by a stepper motor. Another container with a transparent plastic film bottom, which is filled with water, is put on the suspension container. This is to define the target acoustic image plane (the bottom of the transparent plastic film bottom) and reduce the acoustic wave reflection from the top liquid–air interface.

## Supplementary information

Supplementary Information

Description of Additional Supplementary Files

Supplementary Movie 1

Supplementary Movie 2

Supplementary Movie 3

## Data Availability

The datasets generated during and/or analyzed during this study are available from the corresponding author on reasonable request.  Source data are provided with this paper.

## Source data

Source Data

## References

[1] Grier DG (2003). A revolution in optical manipulation. Nature.

[2] Igasaki Y (1999). High efficiency electrically-addressable phase-only spatial light modulator. Optical Rev..

[3] Venediktov VY, Nevskaya G, Tomilin M (2011). Liquid crystals in dynamic holography. Opt. Spectrosc..

[4] Li J (2018). Addressable metasurfaces for dynamic holography and optical information encryption. Sci. Adv..

[5] Liu X (2019). Thermally dependent dynamic meta‐holography using a vanadium dioxide integrated metasurface. Adv. Optical Mater..

[6] Wang Y (2019). 2D broadband beamsteering with large-scale MEMS optical phased array. Optica.

[7] Garret, S. *Understanding Acoustics* (Springer, 2017).

[8] Melde K, Mark AG, Qiu T, Fischer P (2016). Holograms for acoustics. Nature.

[9] Melde K (2018). Acoustic fabrication via the assembly and fusion of particles. Adv. Mater..

[10] Ma, Z. et al. Acoustic holographic cell patterning in a biocompatible hydrogel. *Adv. Mater.* **32**, 1904181 (2019). 10.1002/adma.201904181 31782570

[11] Cox L, Melde K, Croxford A, Fischer P, Drinkwater BW (2019). Acoustic hologram enhanced phased arrays for ultrasonic particle manipulation. Phys. Rev. Appl..

[12] Jiménez-Gambín S, Jiménez N, Benlloch JM, Camarena F (2019). Holograms to focus arbitrary ultrasonic fields through the skull. Phys. Rev. Appl..

[13] Farhadi A, Ho GH, Sawyer DP, Bourdeau RW, Shapiro MG (2019). Ultrasound imaging of gene expression in mammalian cells. Science.

[14] Hu H (2018). Stretchable ultrasonic transducer arrays for three-dimensional imaging on complex surfaces. Sci. Adv..

[15] Drinkwater BW, Wilcox PD (2006). Ultrasonic arrays for non-destructive evaluation: a review. NDT E Int..

[16] Collins DJ, Ma Z, Han J, Ai Y (2017). Continuous micro-vortex-based nanoparticle manipulation via focused surface acoustic waves. Lab a Chip.

[17] Chen M (2017). Observation of metal nanoparticles for acoustic manipulation. Adv. Sci..

[18] Guo F (2015). Controlling cell–cell interactions using surface acoustic waves. Proc. Natl Acad. Sci. USA.

[19] Baudoin M (2019). Folding a focalized acoustical vortex on a flat holographic transducer: miniaturized selective acoustical tweezers. Sci. Adv..

[20] Andrade MA, Bernassau AL, Adamowski JC (2016). Acoustic levitation of a large solid sphere. Appl. Phys. Lett..

[21] Ma G, Fan X, Sheng P, Fink M (2018). Shaping reverberating sound fields with an actively tunable metasurface. Proc. Natl Acad. Sci. USA.

[22] Tian Z (2019). Programmable acoustic metasurfaces. Adv. Funct. Mater..

[23] Szabo, T. L. *Diagnostic Ultrasound Imaging: Inside Out* (Academic Press, 2004).

[24] Marzo A (2015). Holographic acoustic elements for manipulation of levitated objects. Nat. Commun..

[25] Marzo A, Drinkwater BW (2019). Holographic acoustic tweezers. Proc. Natl Acad. Sci. USA.

[26] Hirayama R, Plasencia DM, Masuda N, Subramanian S (2019). A volumetric display for visual, tactile and audio presentation using acoustic trapping. Nature.

[27] Ma G, Sheng P (2016). Acoustic metamaterials: from local resonances to broad horizons. Sci. Adv..

[28] Errico C (2015). Ultrafast ultrasound localization microscopy for deep super-resolution vascular imaging. Nature.

[29] Gong C, Hogan T (2014). CMOS compatible fabrication processes for the digital micromirror device. IEEE J. Electron Devices Soc..

[30] Besse N, Rosset S, Zarate JJ, Shea H (2017). Flexible active skin: large reconfigurable arrays of individually addressed shape memory polymer actuators. Adv. Mater. Technol..

[31] Richter A, Paschew G (2009). Optoelectrothermic control of highly integrated polymer‐based MEMS applied in an artificial skin. Adv. Mater..

[32] Temkin, S. & Temkin, S. *Elements of Acoustics* (Wiley New York, 1981).

[33] Taqieddin A, Nazari R, Rajic L, Alshawabkeh A (2017). Physicochemical hydrodynamics of gas bubbles in two phase electrochemical systems. J. Electrochem. Soc..

[34] Qiu T (2016). Wireless actuation with functional acoustic surfaces. Appl. Phys. Lett..

[35] Patel MV, Nanayakkara IA, Simon MG, Lee AP (2014). Cavity-induced microstreaming for simultaneous on-chip pumping and size-based separation of cells and particles. Lab Chip.

[36] Llewellyn-Jones TM, Drinkwater BW, Trask RS (2016). 3D printed components with ultrasonically arranged microscale structure. Smart Mater. Struct..

[37] Caleap M, Drinkwater BW (2014). Acoustically trapped colloidal crystals that are reconfigurable in real time. Proc. Natl Acad. Sci. USA.

[38] Zhang, X. G. et al. An optically driven digital metasurface for programming electromagnetic functions. *Nat. Electron.* **3**, 165–171 (2020).

[39] Mitrovski SM, Nuzzo RG (2006). A passive microfluidic hydrogen–air fuel cell with exceptional stability and high performance. Lab Chip.

[40] Leroy V (2009). Design and characterization of bubble phononic crystals. Appl. Phys. Lett..

[41] Leroy V (2015). Superabsorption of acoustic waves with bubble metascreens. Phys. Rev. B.

[42] Lanoy M (2017). Acoustic double negativity induced by position correlations within a disordered set of monopolar resonators. Phys. Rev. B.

[43] Leroy V, Chastrette N, Thieury M, Lombard O, Tourin A (2018). Acoustics of bubble arrays: role played by the dipole response of bubbles.. Fluids.

[44] Kruizinga P (2017). Compressive 3D ultrasound imaging using a single sensor. Sci. Adv..

[45] Zimmermann, H. *Silicon Optoelectronic Integrated Circuits* (Springer, 2004).

