# Supplementary material for: Spatial ultrasound modulation by digitally controlling microbubble arrays
Source: Nat Commun. 2020 Sep 10;11:4537. doi: 10.1038/s41467-020-18347-2 (PMC7484750; doi:10.1038/s41467-020-18347-2)
Supplement: Supplementary file 1 — Supplementary Information [file 41467_2020_18347_MOESM1_ESM.pdf]

Supplementary Information

Spatial ultrasound modulation by digitally controlling microbubble arrays

Zhichao Ma, Kai Melde, Athanasios G. Athanassiadis, Michael Schau, Harald Richter, Tian Qiu, Peer Fischer

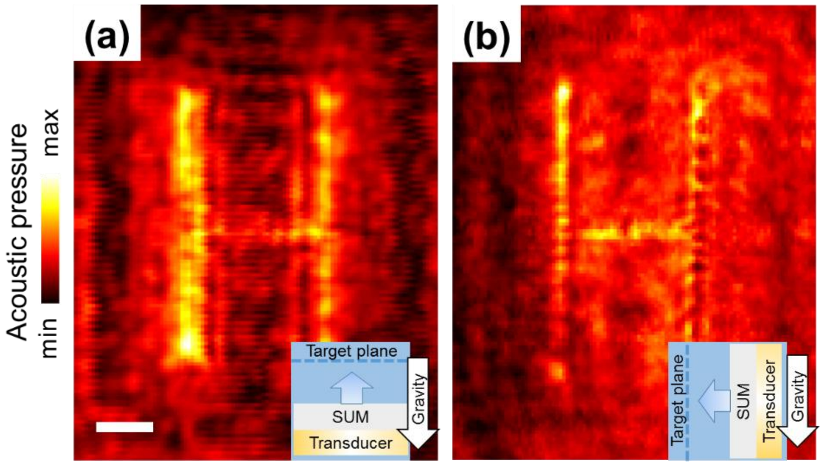

Supplementary Figure 1: SUM in different orientations. The hydrophone scanned "H" shaped acoustic pressure field when the SUM is (a) horizontally and (b) vertically placed, respectively. The ultrasonic modulation by the microbubble pattern is not distorted by the bubble buoyancy due to the microbubbles adherence to the solid surfaces. The device orientations are denoted in the insets. The scale bar is 1 mm.

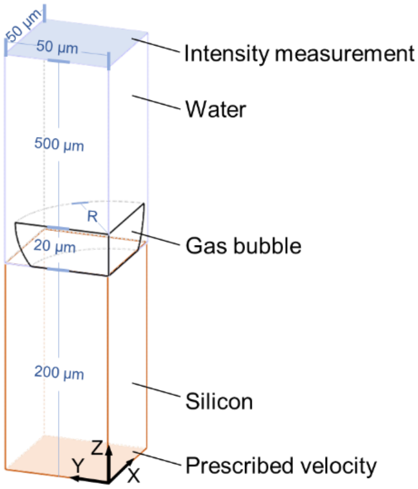

Supplementary Figure 2: Geometry for the numerical calculations. The schematic of the geometry used for the numerical model describing the acoustic transmission through the bubble layer. The geometry represents ¼ of a 100 x 100 µm^2 chip pixel (pad), which is symmetric with respect to the Z-X and the Z-Y planes. The gas bubble domain profile is described by solid surfaces sandwiched by Boolean intersection of an ellipsoid and the 20 µm thick cuboid layer. The bubble cover is then as πR^2, where R is the maximum radius of the bubble domain. The source of the 10 MHz vibration is applied at the bottom surface of silicon domain.

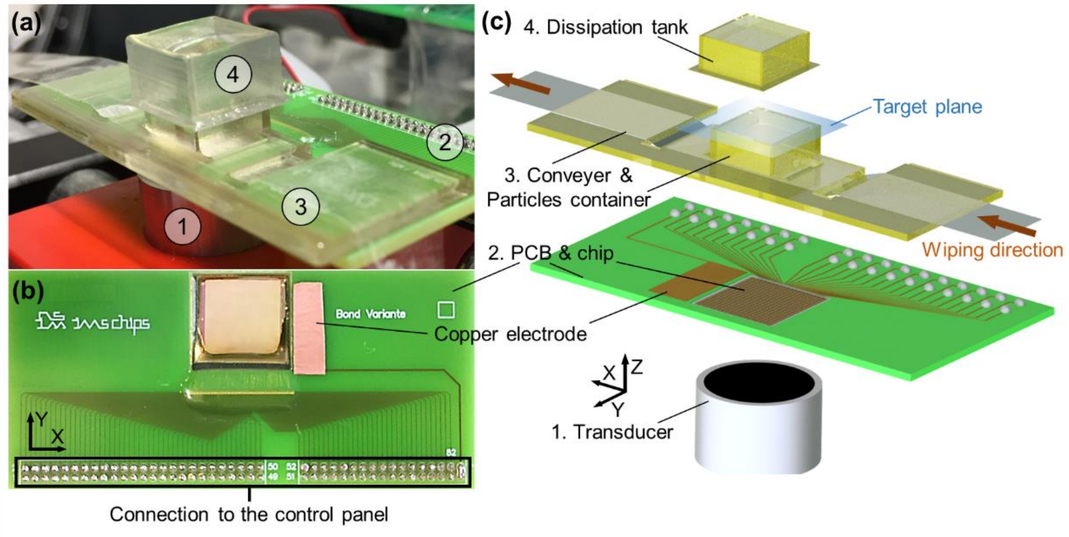

Supplementary Figure 3: Experimental setup. The experimental setup for spatial acoustic modulation. (a) The photograph of an assembled setup. (b) The top view of the CMOS chip mounted on a PCB board. (c) The explosive schematic of the setup. The component labeled with numbers are respectively: (1) acoustic transducer generating the incident plane acoustic waves through the chip; (2) the PCB board and the CMOS chip embedded in it; (3) a 3D printed component containing the particles containing and the conveyer film sandwiched between the particle container and the chip surface; (4) a dissipation tank filled with water that is placed on top of the particle container to reduce the acoustic reflection from the top liquid-air interface.
