# Supplementary material for: Spatial ultrasound modulation by digitally controlling microbubble arrays
Source: Nat Commun. 2020 Sep 10;11:4537. doi: 10.1038/s41467-020-18347-2 (PMC7484750; doi:10.1038/s41467-020-18347-2)
Supplement: Supplementary file 2 — Description of Additional Supplementary Files [file 41467_2020_18347_MOESM2_ESM.pdf]

Description of Additional Supplementary Files

File Name: Supplementary Movie 1
Description: Movie shows the process of spatial ultrasound modulation and dynamic microparticle assembly, including the following three steps: writing of the bubble hologram, acoustic particle assembly and removal of the bubbles.

File Name: Supplementary Movie 2
Description: Movie shows sequential acoustic pressure images at the target plane generated by the spatial ultrasound modulation. The acoustic pressure distribution is acquired with 11 hydrophone scans.

File Name: Supplementary Movie 3
Description: Movie shows the dynamic assembly of microparticles with the spatial ultrasound modulator. Writing the microbubble hologram took around 12 s. At each assembly step, the ultrasound transducer was turned on for 15 s.
